# Prediction of hypertension and diabetes in twin pregnancy using machine learning model based on characteristics at first prenatal visit: national registry study

**DOI:** 10.1002/uog.27710

**Published:** 2025-05-02

**Authors:** H. J. Mustafa, E. Kalafat, S. Prasad, M.‐H. Heydari, R. N. Nunge, A. Khalil

**Affiliations:** ^1^ Department of Obstetrics and Gynecology, Division of Maternal–Fetal Medicine Indiana University School of Medicine Indianapolis IN USA; ^2^ Riley Children and Indiana University Health Fetal Center Indianapolis IN USA; ^3^ Department of Obstetrics and Gynecology Koc University School of Medicine Istanbul Turkey; ^4^ Fetal Medicine Unit, St George's University Hospitals NHS Foundation Trust University of London London UK; ^5^ Non‐Communicable Diseases Research Center, Endocrinology and Metabolism Population Sciences Institute Tehran University of Medical Sciences Tehran Iran; ^6^ Indiana University School of Medicine Indianapolis IN USA; ^7^ Vascular Biology Research Centre, Molecular and Clinical Sciences Research Institute St George's University of London London UK; ^8^ Fetal Medicine Unit Liverpool Women's Hospital Liverpool UK

**Keywords:** artificial intelligence, cross‐sectional study, gestational diabetes mellitus, gestational hypertension, machine learning, pre‐eclampsia, twin pregnancy

## Abstract

**Objective:**

To develop a prediction model for hypertensive disorders of pregnancy (HDP) and gestational diabetes mellitus (GDM) in twin pregnancy using characteristics obtained at the first prenatal visit.

**Methods:**

This was a cross‐sectional study using national live‐birth data in the USA between 2016 and 2021. The association of all prenatal candidate variables with HDP and GDM was tested on univariable and multivariable logistic regression analyses. Prediction models were built with generalized linear models using the logit link function and classification and regression tree (XGboost) machine learning algorithm. Performance was assessed with repeated 2‐fold cross‐validation and the area under the receiver‐operating‐characteristics curve (AUC) was calculated. A *P* value < 0.001 was considered statistically significant.

**Results:**

A total of 707 198 twin pregnancies were included in the HDP analysis and 723 882 twin pregnancies were included in the GDM analysis. The incidence of HDP and GDM increased significantly from 12.6% and 8.1%, respectively, in 2016 to 16.0% and 10.7%, respectively, in 2021. Factors associated with increased odds of HDP in twin pregnancy were maternal age < 20 years or ≥ 35 years, infertility treatment, prepregnancy diabetes mellitus, non‐Hispanic Black race, overweight prepregnancy BMI, prepregnancy obesity and Medicaid as the payment source for delivery (*P* < 0.001 for all). Obesity Class II and III more than doubled the odds of HDP. Factors associated with increased odds of GDM in twin pregnancy were maternal age ≤ 24 years or ≥ 30 years, infertility treatment, prepregnancy hypertension, non‐Hispanic Asian race, maternal birthplace outside the USA and prepregnancy obesity (*P* < 0.001 for all). Maternal age ≥ 30 years, non‐Hispanic Asian race and obesity Class I, II and III more than doubled the odds of GDM. For both HDP and GDM, the performances of the machine learning model and logistic regression model were mostly similar, with negligible differences in the performance domains tested. The mean ± SD AUCs of the final machine learning models for HDP and GDM were 0.620 ± 0.001 and 0.671 ± 0.001, respectively.

**Conclusions:**

The incidence of HDP and GDM in twin pregnancies in the USA is increasing. The predictive accuracy of the machine learning models for HDP and GDM in twin pregnancies was similar to that of the logistic regression models. The models for HDP and GDM had modest predictive performance, were well calibrated and did not have poor fit. © 2024 The Author(s). *Ultrasound in Obstetrics & Gynecology* published by John Wiley & Sons Ltd on behalf of International Society of Ultrasound in Obstetrics and Gynecology.

## INTRODUCTION

Hypertensive disorders of pregnancy (HDP) and gestational diabetes mellitus (GDM) complicate close to 10% and 15%, respectively, of pregnancies worldwide and represent leading causes of maternal and perinatal mortality and morbidity^1^, ^2^. The three major categories of HDP are chronic hypertension, gestational hypertension (GH) and pre‐eclampsia (PE)^3^, ^4^. Although the exact pathophysiology of GH and PE remains unclear, numerous risk factors for these conditions have been identified, including advanced maternal age, history of PE, pre‐existing diabetes mellitus (DM), nulliparity, obesity, renal disorders, immunological disorders and multiple gestation^5^. Known risk factors for GDM include GDM in a previous pregnancy, impaired glucose tolerance, family history of DM (especially in a first‐degree relative), prepregnancy body mass index (BMI) ≥ 30 kg/m^2^, excessive gestational weight gain at 18–24 weeks' gestation, maternal age ≥ 35 years, previous delivery of an infant with birth weight ≥ 4000 g and belonging to a racial/ethnic group with a higher prevalence of Type 2 DM^6^, ^7^.

The incidence of PE and GH is increased in twin compared with singleton pregnancies^8^, ^9^, ^10^, ^11^. This difference can be attributed to an increased maternal inflammatory response, larger placental mass, maternal cardiovascular maladaptation and/or higher levels of placental factors^12^. However, whether multiple gestation constitutes a risk factor for GDM is contested; data on the incidence of GDM in twin compared with singleton pregnancies are inconsistent between studies, possibly because of a lack of adjustment for confounders or because twin pregnancy is not the main exposure^13^, ^14^.

Several studies have reported using machine learning to predict PE in singleton pregnancy^15^, ^16^, ^17^. However, large population‐based studies quantifying the risk of PE in twin pregnancy are scarce and there are no risk‐prediction models specific to twin pregnancy^18^. As twin gestations have been excluded from most studies or, if included, twin pregnancy was not the main exposure, there are limited data on the factors that place a twin pregnancy at risk for GDM and a paucity of prediction models.

This study aimed to assess the incidence and risk factors for HDP and GDM in twin pregnancy, and to build risk‐prediction models using data obtained at the first prenatal visit.

## METHODS

### Study design

This was a cross‐sectional population‐based study using the natality dataset of the USA National Center for Health Statistics and Centers for Disease Control and Prevention (CDC). This dataset includes paternal, maternal, prenatal, labor and obstetric characteristics. It is derived from the national birth registry and uses the US Standard Certificate for live birth. This certificate is completed for every newborn at delivery. The natality dataset does not include any names and the records are anonymous, so the study was exempt from Institutional Review Board appraisal and the need for informed consent was waived.

We included all birth records of twin pregnancies delivered between January 2016 and December 2021. We excluded singletons and higher‐order pregnancies. For HDP analysis, we excluded the records of women with chronic hypertension, and records of women with prepregnancy DM were excluded from GDM analysis.

### Variables and outcomes

The following variables from the CDC dataset obtained at the first prenatal visit were used to develop the prediction models for HDP and GDM: year of delivery, maternal age, maternal birthplace (inside *vs* outside the USA), maternal race/ethnicity, education level, eligibility for the Special Supplemental Nutrition Program for Women, Infants and Children (WIC)^19^, prepregnancy BMI, prepregnancy DM, prepregnancy hypertension, parity, infertility treatment (including use of fertility‐enhancing drugs and *in‐vitro* fertilization (IVF)), periconception cigarette smoking and source of payment for delivery. Variables were selected based on their known clinical relevance to HDP and GDM. Other variables reported in the CDC database that were not included in this study were paternal characteristics, marital status, month of initiation of prenatal care, number of prenatal visits and infection during pregnancy. The CDC natality dataset does not document GH and PE separately, but rather combines them into a single variable (HDP), which we have reflected in our study.

Maternal age was classified as < 20, 20–24, 25–29, 30–34, 35–39 or ≥ 40 years. Women were also categorized as Hispanic or non‐Hispanic, and the latter group was stratified into White, Black, Asian and other (American Indian or Alaska Native (AIAN) and Native Hawaiian and other Pacific Islander (NHOPI)).

The CDC dataset records maternal weight at two timepoints: once before pregnancy and once before birth. Maternal height was also recorded before pregnancy. Hence, prepregnancy BMI was calculated for each record. Individuals were classified into six categories based on their BMI: underweight (< 18.5 kg/m^2^), normal (18.5–24.9 kg/m^2^), overweight (25.0–29.9kg/m^2^), obesity Class I (30.0–34.9 kg/m^2^), obesity Class II (35.0–39.9 kg/m^2^) and obesity Class III (≥ 40 kg/m^2^).

The main study outcomes were the development of HDP and GDM.

### Statistical analysis

The incidence of HDP and GDM in twin pregnancies was calculated for each year from 2016 to 2021. Binomial regression with a complementary log–log link was employed to estimate incidence rate ratios with changing year^20^. The association of all candidate variables in the CDC dataset (maternal age, parity, infertility treatment, education level, prepregnancy DM (for HDP) or hypertension (for GDM), periconception cigarette smoking, maternal race/ethnicity, maternal birthplace, prepregnancy BMI, payment source for delivery and eligibility for WIC) with HDP and GDM was tested on univariable and adjusted multivariable logistic regression analyses. These results are reported as odds ratios (OR) and adjusted odds ratios (aOR), respectively, with 95% CI.

Prediction models were built with generalized linear models using the logit link function and classification and regression tree (XGboost) machine learning algorithm. To identify the most parsimonious model that did not sacrifice performance, all variables were included initially and then eliminated in a stepwise manner. Shapley additive explanation plots were generated for the full model to observe the individual impact of all variables, which also guided the elimination process. To prevent overfitting and allow for realistic assessment of model performance, repeated 2‐fold cross‐validation was employed. The dataset was split in a 1:1 ratio for training and testing over 1000 iterations and training parameters were fine‐tuned to try to avoid the predictive performance shrinkage exceeding 2% between the training and validation sets. The performance metrics considered were area under the receiver‐operating‐characteristics curve (AUC), AUC shrinkage between training and validation sets, calibration intercept, calibration slope and Brier score. The most parsimonious model that did not sacrifice overall performance was selected as the final model. The performance of the final model was reported with calibration curves, positive and negative predictive values at specified cut‐off points and decision curves^21^.

The dataset was checked for the presence of implausible values and extreme outliers. If deemed erroneous, such implausible values and outliers were treated as missing. Missingness of data was minimal (< 3%) and was handled with imputation. Missingness was assumed to occur at random and values were imputed with multiple chained equations. The predictor matrix was formed using all variables (including the outcome) with a correlation coefficient greater than 0.10 with the imputed variable. Finally, the imputation convergence was checked and the density of the imputed variables was cross‐checked against the observed values.

Statistical analysis was performed using RStudio (RStudio Inc., Boston, MA, USA). A *P*‐value < 0.001 was considered statistically significant due to the large number of available observations. An online calculator was designed and deployed using Shiny Applications (Posit Software, Boston, MA, USA).

## RESULTS

### Incidence of hypertensive disorders of pregnancy and gestational diabetes mellitus

Following exclusions, 707 198 twin pregnancies were included in the HDP analysis and 723 882 twin pregnancies were included in the GDM analysis. The incidence of HDP and GDM increased significantly from 12.6% and 8.1%, respectively, in 2016 to 16.0% and 10.7%, respectively, in 2021 (Table 1).

**Table 1:** Annual incidence of hypertensive disorders of pregnancy (HDP) (gestational hypertension or pre‐eclampsia) and gestational diabetes mellitus (GDM) in twin pregnancies delivering in USA between 2016 and 2021

Year	Cases (*n*)	Pregnancies (*n*)	Incidence (%)	IRR (95% CI)*	*P* *
HDP					
2016	16 125	128 337	12.6	Reference	—
2017	16 150	124 731	12.9	1.03 (1.01–1.05)	0.011
2018	16 789	119 933	14.0	1.11 (1.08–1.13)	< 0.001
2019	17 113	116 321	14.7	1.16 (1.13–1.18)	< 0.001
2020	16 583	108 329	15.3	1.20 (1.18–1.23)	< 0.001
2021	17 532	109 547	16.0	1.25 (1.23–1.28)	< 0.001
GDM					
2016	10 568	130 700	8.1	Reference	—
2017	10 834	127 198	8.5	1.05 (1.02–1.08)	< 0.001
2018	11 251	122 540	9.2	1.13 (1.10–1.16)	< 0.001
2019	10 874	119 351	9.1	1.12 (1.09–1.15)	< 0.001
2020	10 987	111 254	9.9	1.21 (1.18–1.24)	< 0.001
2021	12 108	112 839	10.7	1.31 (1.28–1.35)	< 0.001

* Binomial regression with complementary log–log link.

IRR, incidence rate ratio.

### Risk of hypertensive disorders of pregnancy in twin pregnancy

Univariable and adjusted multivariable logistic regression analyses for the odds of HDP in twin pregnancy are summarized in Table 2. Factors associated with increased odds of HDP were: maternal age < 20 years (aOR, 1.21 (95% CI, 1.15–1.26); *P* < 0.001) or ≥ 35 years (35–39 years: (aOR, 1.11 (95% CI, 1.08–1.13); *P* < 0.001); ≥ 40 years: (aOR, 1.35 (95% CI, 1.31–1.39); *P* < 0.001)); infertility treatment with fertility‐enhancing drugs (aOR, 1.64 (95% CI, 1.60–1.68); *P* < 0.001) or IVF (aOR, 1.34 (95% CI, 1.30–1.38); *P* < 0.001); prepregnancy DM (aOR, 1.92 (95% CI, 1.81–2.04); *P* < 0.001); non‐Hispanic Black race (aOR, 1.05 (95% CI, 1.03–1.07); *P* < 0.001); maternal overweight prepregnancy BMI (aOR, 1.36 (95% CI, 1.33–1.38); *P* < 0.001) or prepregnancy obesity (Class I: (aOR, 1.73 (95% CI, 1.70–1.76); *P* < 0.001); Class II: (aOR, 2.09 (95% CI, 2.04–2.14); *P* < 0.001); Class III: (aOR, 2.80 (95% CI, 2.74–2.88); *P* < 0.001)); and Medicaid as the payment source for delivery (aOR, 1.08 (95% CI, 1.06–1.11); *P* < 0.001).

**Table 2:** Univariable and adjusted multivariable logistic models of population characteristics in twin pregnancies, according to presence or absence of hypertensive disorders of pregnancy (HDP)

Variable	No HDP	HDP	OR (95% CI)	*P*	aOR (95% CI)	*P*
Maternal age						
25–29 years	163 924 (86.3)	25 943 (13.7)	Reference	—	Reference	—
< 20 years	14 528 (85.1)	2540 (14.9)	1.10 (1.06–1.15)	< 0.001	1.21 (1.15–1.26)	< 0.001
20–24 years	84 822 (86.3)	13 452 (13.7)	1.00 (0.98–1.02)	0.856	1.02 (1.00–1.05)	0.046
30–34 years	198 687 (86.2)	31 756 (13.8)	1.01 (0.99–1.03)	0.274	1.01 (1.00–1.03)	0.136
35–39 years	116 155 (85.2)	20 242 (14.8)	1.10 (1.08–1.12)	< 0.001	1.11 (1.08–1.13)	< 0.001
≥ 40 years	28 790 (81.9)	6359 (18.1)	1.40 (1.35–1.44)	< 0.001	1.35 (1.31–1.39)	< 0.001
Parity						
Nulliparous	137 184 (81.7)	30 754 (18.3)	Reference	—	Reference	—
Parous	469 722 (87.1)	69 538 (12.9)	0.66 (0.65–0.67)	< 0.001	0.66 (0.65–0.68)	< 0.001
Infertility treatment						
None	540 162 (86.6)	83 474 (13.4)	Reference	—	Reference	—
Fertility‐enhancing drugs	39 860 (78.9)	10 633 (21.1)	1.73 (1.69–1.77)	< 0.001	1.64 (1.60–1.68)	< 0.001
IVF	26 884 (81.3)	6185 (18.7)	1.49 (1.45–1.53)	< 0.001	1.34 (1.30–1.38)	< 0.001
Education level						
≥ 12^th^ grade	549 226 (85.5)	93 036 (14.5)	Reference	—	Reference	—
< 12^th^ grade	57 680 (88.8)	7256 (11.2)	0.74 (0.72–0.76)	< 0.001	0.90 (0.87–0.92)	< 0.001
Prepregnancy DM						
No	602 453 (85.9)	98 636 (14.1)	Reference	—	Reference	—
Yes	4453 (72.9)	1656 (27.1)	2.27 (2.15–2.40)	< 0.001	1.92 (1.81–2.04)	< 0.001
Periconception cigarette smoking						
No	559 283 (85.7)	93 052 (14.3)	Reference		Reference	
Yes	12 417 (85.3)	2146 (14.7)	1.04 (0.99–1.09)	0.108	1.07 (1.02–1.12)	0.005
Maternal race/ethnicity						
NH White	331 629 (85.1)	58 257 (14.9)	Reference	—	Reference	—
NH Black	107 024 (84.7)	19 370 (15.3)	1.03 (1.01–1.05)	0.001	1.05 (1.03–1.07)	< 0.001
NH Asian	34 708 (89.6)	4026 (10.4)	0.66 (0.64–0.68)	< 0.001	0.80 (0.77–0.83)	< 0.001
Hispanic	114 453 (88.1)	15 507 (11.9)	0.77 (0.76–0.79)	< 0.001	0.88 (0.86–0.90)	< 0.001
NH other	19 092 (85.9)	3132 (14.1)	0.93 (0.90–0.97)	0.001	0.95 (0.91–0.99)	0.009
Maternal birthplace						
Inside USA	482 935 (85.0)	85 431 (15.0)	Reference	—	Reference	—
Outside USA	123 971 (89.3)	14 861 (10.7)	0.68 (0.67–0.69)	< 0.001	0.80 (0.78–0.81)	< 0.001
Prepregnancy BMI						
Normal	243 052 (89.2)	29 311 (10.8)	Reference	—	Reference	—
Underweight	15 237 (91.4)	1429 (8.6)	0.78 (0.74–0.82)	< 0.001	0.82 (0.78–0.87)	< 0.001
Overweight	164 239 (86.3)	26 072 (13.7)	1.32 (1.29–1.34)	< 0.001	1.36 (1.33–1.38)	< 0.001
Obesity Class I	97 549 (83.4)	19 457 (16.6)	1.65 (1.62–1.69)	< 0.001	1.73 (1.70–1.76)	< 0.001
Obesity Class II	50 530 (80.4)	12 305 (19.6)	2.02 (1.97–2.07)	< 0.001	2.09 (2.04–2.14)	< 0.001
Obesity Class III	36 299 (75.6)	11 718 (24.4)	2.68 (2.61–2.74)	< 0.001	2.80 (2.74–2.88)	< 0.001
Payment source for delivery						
Self‐pay	231 136 (86.9)	34 698 (13.1)	Reference	—	Reference	—
Medicaid	331 406 (84.7)	59 921 (15.3)	1.20 (1.19–1.22)	< 0.001	1.08 (1.06–1.11)	< 0.001
Private insurance	19 383 (90.8)	1969 (9.2)	0.68 (0.64–0.71)	< 0.001	0.80 (0.77–0.85)	< 0.001
Other	21 526 (87.0)	3205 (13.0)	0.99 (0.95–1.03)	0.678	0.98 (0.94–1.02)	0.392
WIC program						
Ineligible	402 424 (85.4)	68 960 (14.6)	Reference	—	Reference	—
Eligible	204 482 (86.7)	31 332 (13.3)	0.89 (0.88–0.91)	< 0.001	0.96 (0.94–0.97)	< 0.001

Data are given as *n* (%), unless stated otherwise.

aOR, adjusted odds ratio; BMI, body mass index; DM, diabetes mellitus; IVF, *in‐vitro* fertilization; NH, non‐Hispanic; OR, odds ratio; WIC, Special Supplemental Nutrition Program for Women, Infants, and Children.

Factors associated with reduced odds of HDP in twin pregnancy were: parity ≥ 1 (aOR, 0.66 (95% CI, 0.65–0.68); *P* < 0.001); non‐Hispanic Asian race (aOR, 0.80 (95% CI, 0.77–0.83); *P* < 0.001) and Hispanic race (aOR, 0.88 (95% CI, 0.86–0.90); *P* < 0.001); maternal underweight prepregnancy BMI (aOR, 0.82 (95% CI, 0.78–0.87); *P* < 0.001); maternal birthplace outside the USA (aOR, 0.80 (95% CI, 0.78–0.81); *P* < 0.001); private insurance as the payment source for delivery (aOR, 0.80 (95% CI, 0.77–0.85); *P* < 0.001); and eligibility for the WIC program (aOR, 0.96 (95% CI, 0.94–0.97); *P* < 0.001).

### Risk of gestational diabetes mellitus in twin pregnancy

Univariable and adjusted multivariable logistic regression analyses for the odds of GDM in twin pregnancy are summarized in Table 3. Factors associated with an increased odds of GDM in twin pregnancy were: maternal age ≤ 24 years (< 20 years: (aOR, 1.33 (95% CI, 1.22–1.46); *P* < 0.001); 20–24 years: (aOR, 2.01 (95% CI, 1.84–2.20); *P* < 0.001)) or ≥ 30 years (30–34 years: (aOR, 2.69 (95% CI, 2.47–2.94); *P* < 0.001); 35–39 years: (aOR, 3.39 (95% CI, 3.10–3.71); *P* < 0.001); ≥ 40 years: (aOR, 4.00 (95% CI, 3.65–4.39); *P* < 0.001)); infertility treatment with fertility‐enhancing drugs (aOR, 1.29 (95% CI, 1.26–1.33); *P* < 0.001) or IVF (aOR, 1.33 (95% CI, 1.28–1.37); *P* < 0.001); prepregnancy hypertension (aOR, 1.69 (95% CI, 1.63–1.75); *P* < 0.001); non‐Hispanic Asian race (aOR, 2.09 (95% CI, 2.03–2.16); *P* < 0.001); maternal birthplace outside the USA (aOR, 1.39 (95% CI, 1.36–1.42); *P* < 0.001); maternal underweight prepregnancy BMI (aOR, 1.17 (95% CI, 1.09–1.25); *P* < 0.001), overweight prepregnancy BMI (aOR, 1.79 (95% CI, 1.67–1.93); *P* < 0.001) or prepregnancy obesity (Class I: (aOR, 2.56 (95% CI, 2.38–2.75); *P* < 0.001); Class II: (aOR, 3.46 (95% CI, 3.21–3.72); *P* < 0.001); Class III: (aOR, 4.35 (95% CI, 4.05–4.69); *P* < 0.001)); private insurance as the payment source for delivery (aOR, 1.07 (95% CI, 1.04–1.09); *P* < 0.001) and eligibility for the WIC program (aOR, 1.10 (95% CI, 1.08–1.13); *P* < 0.001).

**Table 3:** Univariable and adjusted multivariable logistic models of population characteristics in twin pregnancies, according to presence or absence of gestational diabetes mellitus (GDM)

Variable	No GDM	GDM	OR (95% CI)	*P*	aOR (95% CI)	*P*
Maternal age						
25–29 years	16 724 (96.8)	544 (3.2)	Reference	—	Reference	—
< 20 years	95 188 (95.2)	4766 (4.8)	1.54 (1.41–1.69)	< 0.001	1.33 (1.22–1.46)	< 0.001
20–24 years	179 469 (92.6)	14 442 (7.4)	2.47 (2.27–2.70)	< 0.001	2.01 (1.84–2.20)	< 0.001
30–34 years	212 439 (90.1)	23 443 (9.9)	3.39 (3.11–3.70)	< 0.001	2.69 (2.47–2.94)	< 0.001
35–39 years	122 584 (87.4)	17 715 (12.6)	4.44 (4.08–4.85)	< 0.001	3.39 (3.10–3.71)	< 0.001
≥ 40 years	30 856 (84.4)	5712 (15.6)	5.69 (5.21–6.23)	< 0.001	4.00 (3.65–4.39)	< 0.001
Parity						
Nulliparous	156 017 (90.9)	15 624 (9.1)	Reference	—	Reference	—
Parous	501 243 (90.8)	50 998 (9.2)	1.02 (1.00–1.04)	0.098	0.97 (0.95–0.99)	0.009
Infertility treatment						
None	583 126 (91.4)	54 895 (8.6)	Reference	—	Reference	—
Fertility‐enhancing drugs	44 694 (86.0)	7278 (14.0)	1.73 (1.68–1.78)	< 0.001	1.29 (1.26–1.33)	< 0.001
IVF	29 440 (86.9)	4449 (13.1)	1.61 (1.55–1.66)	< 0.001	1.33 (1.28–1.37)	< 0.001
Education level						
≥ 12^th^ grade	596 473 (90.7)	61 155 (9.3)	Reference	—	Reference	—
< 12^th^ grade	60 787 (91.7)	5467 (8.3)	0.88 (0.85–0.90)	< 0.001	1.00 (0.97–1.03)	0.910
Prepregnancy hypertension						
No	638 579 (91.1)	62 510 (8.9)	Reference	—	Reference	—
Yes	18 681 (82.0)	4112 (18.0)	2.25 (2.17–2.33)	< 0.001	1.69 (1.63–1.75)	< 0.001
Periconception cigarette smoking						
Non‐smoker	605 113 (90.7)	62 062 (9.3)	Reference	—	Reference	—
1–5 per day	13 931 (92.2)	1185 (7.8)	0.83 (0.78–0.88)	< 0.001	1.01 (0.95–1.07)	0.722
6–10 per day	17 380 (91.8)	1553 (8.2)	0.87 (0.83–0.92)	< 0.001	1.03 (0.97–1.08)	0.359
11–20 per day	17 609 (92.0)	1540 (8.0)	0.85 (0.81–0.90)	< 0.001	1.00 (0.95–1.06)	0.976
21–40 per day	3227 (92.0)	282 (8.0)	0.85 (0.75–0.96)	0.010	1.01 (0.89–1.15)	0.824
Maternal race/ethnicity						
NH White	362 493 (91.0)	35 791 (9.0)	Reference	—	Reference	—
NH Black	123 906 (93.5)	8657 (6.5)	0.71 (0.69–0.73)	< 0.001	0.63 (0.62–0.65)	< 0.001
NH Asian	31 679 (81.2)	7313 (18.8)	2.34 (2.27–2.40)	< 0.001	2.09 (2.03–2.16)	< 0.001
Hispanic	118 487 (90.3)	12 795 (9.7)	1.09 (1.07–1.12)	< 0.001	1.00 (0.97–1.02)	0.799
NH other	20 695 (90.9)	2066 (9.1)	1.01 (0.96–1.06)	0.642	1.01 (0.96–1.06)	0.647
Maternal birthplace						
Inside USA	534 677 (91.6)	48 962 (8.4)	Reference	—	Reference	—
Outside USA	122 583 (87.4)	17 660 (12.6)	1.57 (1.54–1.60)	< 0.001	1.39 (1.36–1.42)	< 0.001
Prepregnancy BMI						
Normal	15 868 (94.7)	883 (5.3)	Reference	—	Reference	—
Underweight	257 014 (93.7)	17 314 (6.3)	1.21 (1.13–1.30)	< 0.001	1.17 (1.09–1.25)	< 0.001
Overweight	176 734 (91.2)	17 036 (8.8)	1.73 (1.62–1.86)	< 0.001	1.79 (1.67–1.93)	< 0.001
Obesity Class I	107 012 (88.7)	13 689 (11.3)	2.30 (2.14–2.47)	< 0.001	2.56 (2.38–2.75)	< 0.001
Obesity Class II	56 633 (86.1)	9145 (13.9)	2.90 (2.70–3.12)	< 0.001	3.46 (3.21–3.72)	< 0.001
Obesity Class III	43 999 (83.7)	8555 (16.3)	3.49 (3.25–3.76)	< 0.001	4.35 (4.05–4.69)	< 0.001
Payment source for delivery						
Medicaid	250 826 (91.8)	22 269 (8.2)	Reference	—	Reference	—
Private insurance	359 424 (89.9)	40 466 (10.1)	1.27 (1.25–1.29)	< 0.001	1.07 (1.04–1.09)	< 0.001
Self‐pay	20 147 (93.4)	1416 (6.6)	0.79 (0.75–0.84)	< 0.001	0.65 (0.61–0.69)	< 0.001
WIC program						
Ineligible	436 171 (90.5)	45 627 (9.5)	Reference	—	Reference	—
Eligible	221 089 (91.3)	20 995 (8.7)	0.91 (0.89–0.92)	< 0.001	1.10 (1.08–1.13)	< 0.001

Data are given as *n* (%), unless stated otherwise.

aOR, adjusted odds ratio; BMI, body mass index; IVF, *in‐vitro* fertilization; NH, non‐Hispanic; OR, odds ratio; WIC, Special Supplemental Nutrition Program for Woman, Infants, and Children.

Factors associated with reduced odds of GDM in twin pregnancy were non‐Hispanic Black race (aOR, 0.63 (95% CI, 0.62–0.65); *P* < 0.001) and self‐pay as the payment source for delivery (aOR, 0.65 (95% CI, 0.61–0.69); *P* < 0.001).

### Risk‐prediction models for hypertensive disorders of pregnancy in twin pregnancy

**Figure 1:**
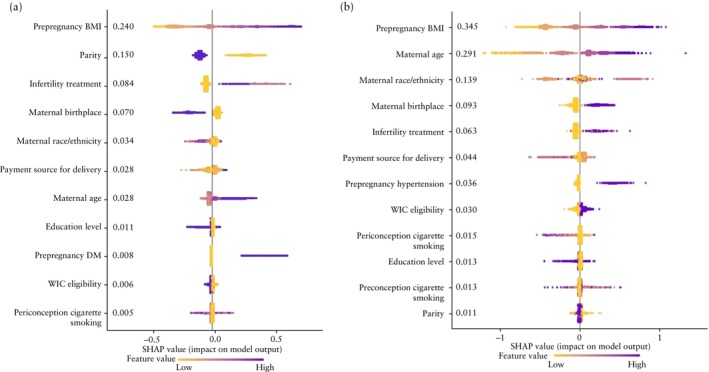
Shapley additive explanation (SHAP) plots showing importance of each variable to model output for prediction of hypertensive disorders of pregnancy (a) and gestational diabetes mellitus (b) in twin pregnancy. BMI, body mass index; DM, diabetes mellitus; WIC, Special Supplemental Nutrition Program for Women, Infants, and Children.

Initially, all variables in the candidate pool were used to train the largest model for HDP and rank the importance of each feature. The five most important variables for the prediction of HDP in twin pregnancy were prepregnancy BMI, parity, infertility treatment, maternal birthplace and maternal race/ethnicity (Figure 1a). Among the tested models, the most parsimonious one that did not sacrifice performance included maternal age, parity, IVF, prepregnancy DM and prepregnancy BMI (Table 4). The mean ± SD AUC, calibration intercept and slope of the final machine learning model for HDP were 0.620 ± 0.001, 0.061 ± 0.027 and 1.074 ± 0.015, respectively. The corresponding values for the logistic regression model incorporating the same variables were 0.617 ± 0.001, 0.002 ± 0.028 and 1.001 ± 0.015.

**Table 4:** Internal validation performance metrics for models trained with classification and regression tree (XGboost) and logistic regression for development of hypertensive disorders of pregnancy in twin pregnancy

Model	AUC	AUC shrinkage* (%)	Calibration intercept	Calibration slope	Brier score
Classification and regression tree (XGboost)					
Maternal age, parity, IVF, education level, prepregnancy DM, periconception cigarette smoking, maternal race/ethnicity, maternal birthplace, prepregnancy BMI, payment source for delivery, WIC eligibility	0.629 ± 0.001	0.42 ± 0.29	0.176 ± 0.029	1.143 ± 0.018	0.11844 ± 0.00027
Maternal age, parity, IVF, prepregnancy DM, prepregnancy BMI†	0.620 ± 0.001	0.23 ± 0.28	0.061 ± 0.027	1.074 ± 0.015	0.11884 ± 0.00028
Maternal age, parity, IVF, prepregnancy BMI	0.618 ± 0.001	0.16 ± 0.29	0.055 ± 0.026	1.069 ± 0.015	0.11896 ± 0.00028
Parity, IVF, prepregnancy BMI	0.616 ± 0.001	0.12 ± 0.32	0.034 ± 0.033	1.058 ± 0.018	0.11899 ± 0.00024
Random selection	0.501 ± 0.001	7.05 ± 0.51	−1.71 ± 0.192	0.052 ± 0.111	0.12171 ± 0.00025
Logistic regression					
Maternal age, parity, IVF, education level, prepregnancy DM, periconception cigarette smoking, maternal race/ethnicity, maternal birthplace, prepregnancy BMI, payment source for delivery, WIC eligibility	0.623 ± 0.001	−0.01 ± 0.33	0.001 ± 0.031	1.000 ± 0.017	0.11871 ± 0.00030
Maternal age, parity, IVF, prepregnancy DM, prepregnancy BMI†	0.617 ± 0.001	−0.02 ± 0.30	0.002 ± 0.028	1.001 ± 0.015	0.11899 ± 0.00027
Maternal age, parity, IVF, prepregnancy BMI	0.615 ± 0.001	−0.07 ± 0.32	0.006 ± 0.030	1.004 ± 0.018	0.11905 ± 0.00031
Parity, IVF, prepregnancy BMI	0.614 ± 0.001	0.06 ± 0.35	−0.007 ± 0.033	0.997 ± 0.019	0.11913 ± 0.00027
Random selection	0.499 ± 0.001	0.54 ± 0.27	−3.226 ± 1.002	−0.792 ± 0.557	0.12167 ± 0.00031

Data are given as mean ± SD.

* Between training and validation sets.

† Final model.

AUC, area under receiver‐operating‐characteristics curve; BMI, body mass index; DM, diabetes mellitus; IVF, *in‐vitro* fertilization; WIC, Special Supplemental Nutrition Program for Woman, Infants, and Children.

**Figure 2:**
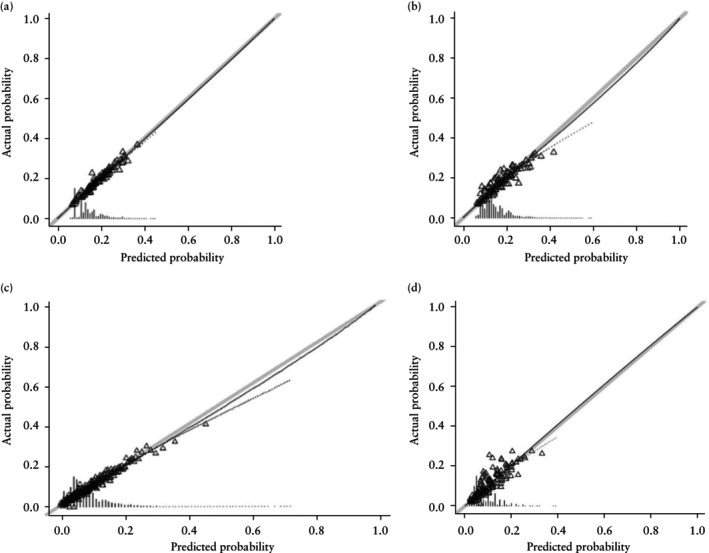
Calibration plots for classification and regression tree prediction models (a,c) and logistic regression prediction models (b,d) for prediction of hypertensive disorders of pregnancy (a,b) and gestational diabetes mellitus (c,d) in twin pregnancy from a single validation set. 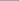, ideal; 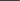, logistic calibration; 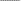, non‐parametric; 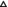, grouped observation.

**Figure 3:**
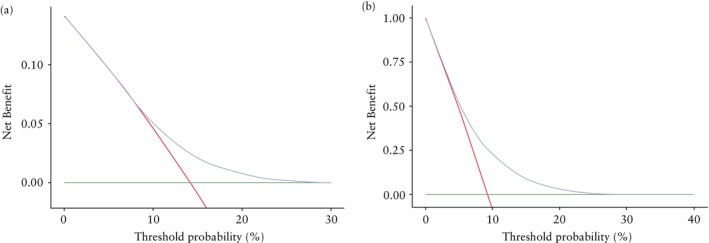
Decision curves for final models (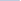) for prediction of hypertensive disorders of pregnancy (a) and gestational diabetes mellitus (b) in twin pregnancy. 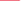, Treat all; 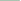, treat none.

The performance of the machine learning and logistic regression models was mostly similar, with negligible differences in the performance domains tested (Table 4). Both models were well calibrated and had no predicted probability regions over which they under‐ or overestimated the risk of HDP for the majority of the population (Table 4, Figure 2a,b). The risk deciles and predictive accuracy of different probability cut‐offs were assessed (Table 5). Estimated risk corresponded to actual observed rate of HDP in all categories. Decision curve analysis showed that the final machine learning model had higher Net Benefit compared with the treat‐all approach for predicted risks over 10%, with a positive predictive value of 14.3% and a negative predictive value of 93.0% (Table 5, Figure 3a).

**Table 5:** Size of risk groups, observed outcome rate within risk groups and positive (PPV) and negative (NPV) predictive values, according to final machine learning model for prediction of hypertensive disorders of pregnancy (HDP) in twin pregnancy

Risk group	Patients (*n* = 707 198)*	HDP (*n*)	Observed HDP rate (%)	PPV (% (95% CI))	NPV (% (95% CI))	Cut‐off used
≤ 10%	10 260 (1.5)	720	7.0	—	—	—
> 10% to ≤ 20%	586 183 (82.9)	72 940	12.4	14.3 (14.2–14.4)	93.0 (92.5–93.5)	> 10%
> 20% to ≤ 30%	108 158 (15.3)	25 700	23.8	24.1 (23.8–24.3)	87.7 (87.6–87.7)	> 20%
> 30%	2597 (0.4)	932	35.9	35.9 (34.0–37.8)	85.9 (85.8–86.0)	> 30%

* Data are given as *n* (%).

An online calculator was developed for external use and validation (https://epsilonkappa.shinyapps.io/HYPERTIP/).

### Risk‐prediction models for gestational diabetes mellitus in twin pregnancy

Initially, all variables in the candidate pool were used to train the largest model for GDM and rank the importance of each feature. The five variables with the highest impact on the predicted probabilities of GDM were prepregnancy BMI, maternal age, maternal race/ethnicity, maternal birthplace and infertility treatment (Figure 1b). Among the tested models, the most parsimonious one that did not sacrifice performance included maternal age, maternal race/ethnicity and prepregnancy BMI (Table 6). The mean ± SD AUC, calibration intercept and slope of the final machine learning model for GDM were 0.671 ± 0.001, − 0.011 ± 0.026 and 1.003 ± 0.013, respectively. The corresponding values for the logistic regression model incorporating the same variables were 0.650 ± 0.001, 0.003 ± 0.032 and 1.001 ± 0.014.

**Table 6:** Internal validation performance metrics for models trained with classification and regression tree (XGboost) and logistic regression for development of gestational diabetes mellitus in twin pregnancy

Model	AUC	AUC shrinkage* (%)	Calibration intercept	Calibration slope	Brier score
Classification and regression tree (XGboost)					
Maternal age, parity, IVF, education level, prepregnancy hypertension, periconception cigarette smoking, maternal race/ethnicity, maternal birthplace, prepregnancy BMI, payment source for delivery, WIC eligibility	0.682 ± 0.001	2.75 ± 0.30	−0.037 ± 0.027	0.993 ± 0.013	0.08015 ± 0.00026
Maternal age, IVF, prepregnancy hypertension, maternal race/ethnicity, maternal birthplace, prepregnancy BMI	0.677 ± 0.001	0.93 ± 0.28	−0.053 ± 0.027	0.984 ± 0.011	0.08041 ± 0.00029
Maternal age, maternal race/ethnicity, maternal birthplace, prepregnancy BMI	0.674 ± 0.001	0.41 ± 0.40	−0.015 ± 0.035	1.002 ± 0.016	0.08057 ± 0.00027
Maternal age, maternal race/ethnicity, prepregnancy BMI†	0.671 ± 0.001	0.37 ± 0.27	−0.011 ± 0.026	1.003 ± 0.013	0.08067 ± 0.00024
Maternal age, prepregnancy BMI	0.648 ± 0.001	0.02 ± 0.31	0.020 ± 0.036	1.016 ± 0.015	0.08153 ± 0.00027
Random selection	0.500 ± 0.002	22.86 ± 1.11	−2.302 ± 0.123	−0.005 ± 0.054	0.08366 ± 0.00031
Logistic regression					
Maternal age, parity, IVF, education level, prepregnancy hypertension, periconception cigarette smoking, maternal race/ethnicity, maternal birthplace, prepregnancy BMI, payment source for delivery, WIC eligibility	0.664 ± 0.001	0.02 ± 0.33	0.000 ± 0.034	0.999 ± 0.015	0.08108 ± 0.00024
Maternal age, IVF, prepregnancy hypertension, maternal race/ethnicity, maternal birthplace, prepregnancy BMI	0.663 ± 0.001	0.01 ± 0.35	0.000 ± 0.036	1.000 ± 0.016	0.08110 ± 0.00024
Maternal age, maternal race/ethnicity, maternal birthplace, prepregnancy BMI	0.656 ± 0.001	0.02 ± 0.31	0.002 ± 0.031	1.001 ± 0.014	0.08126 ± 0.00027
Maternal age, maternal race/ethnicity, prepregnancy BMI†	0.650 ± 0.001	−0.01 ± 0.31	0.003 ± 0.032	1.001 ± 0.014	0.08149 ± 0.00024
Maternal age, prepregnancy BMI	0.648 ± 0.001	−0.07 ± 0.35	0.007 ± 0.035	1.003 ± 0.015	0.08157 ± 0.00029
Random selection	0.502 ± 0.001	0.36 ± 0.29	−1.058 ± 0.856	0.538 ± 0.375	0.08354 ± 0.00034

Data are given as mean ± SD.

* Between training and validation sets.

† Final model.

AUC, area under receiver‐operating‐characteristics curve, BMI, body mass index; IVF, *in‐vitro* fertilization; WIC, Special Supplemental Nutrition Program for Woman, Infants, and Children.

The performance of the machine learning and logistic regression models was mostly similar, with negligible differences in the performance domains tested (Table 6). Both models were well calibrated and had no predicted probability regions over which they under‐ or overestimated the risk of GDM for the majority of the population (Table 6, Figure 2c,d). Predictive accuracy was assessed for different categories of estimated risk, from ≤ 5% to > 25% (Table 7). Estimated risk corresponded to actual observed rate of GDM in all categories. Decision curve analysis showed that the final machine learning model had higher Net Benefit compared with the treat‐all approach for predicted risks over 10%, with a positive predictive value of 15.4% and a negative predictive value of 94.0% (Table 7, Figure 3b).

**Table 7:** Size of risk groups, observed outcome rate within risk groups and positive (PPV) and negative (NPV) predictive values, according to final machine learning model for prediction of gestational diabetes mellitus (GDM) in twin pregnancy

Risk group	Patients (*n* = 723 882)*	GDM (*n*)	Observed GDM rate (%)	PPV (% (95% CI))	NPV (% (95% CI))	Cut‐off used
≤ 5%	157 172 (21.7)	5778	3.7	—	—	—
> 5% to ≤ 10%	323 136 (44.6)	23 240	7.2	10.7 (10.7–10.8)	96.3 (96.2–96.4)	> 5%
> 10% to ≤ 15%	124 164 (17.2)	14 890	12.0	15.4 (15.3–15.6)	94.0 (93.9–94.0)	> 10%
> 15% to ≤ 20%	76 874 (10.6)	12 878	16.8	19.0 (18.8–19.3)	92.7 (92.7–92.8)	> 15%
> 20% to ≤ 25%	35 950 (5.0)	7919	22.0	23.1 (22.7–23.5)	91.7 (91.6–91.7)	> 20%
> 25%	6586 (0.9)	1917	29.1	29.1 (28.0–30.2)	91.0 (90.9–91.1)	> 25%

* Data are given as *n* (%).

An online calculator was developed for external use and validation (https://epsilonkappa.shinyapps.io/BRAD‐GDT/).

## DISCUSSION

### Summary of key findings

In this large cross‐sectional population‐based study using live‐birth data from the USA between 2016 and 2021, we report that the incidence of HDP and GDM in twin pregnancies increased from 2016 to 2021. Factors that more than doubled the risk of HDP and were at the top of the Shapley additive explanation graph were prepregnancy obesity Class II and III, whereas, for the development of GDM, prepregnancy obesity Class I, II and III, maternal age ≥ 30 years and non‐Hispanic Asian race were the strongest risk factors. The predictive accuracy of the machine learning models for HDP and GDM in twin gestations was similar to that of the logistic regression models, based on data from the first prenatal visit. The models for HDP and GDM had modest predictive performance, were well calibrated and did not have poor fit.

### Comparison with literature

The American College of Obstetricians and Gynecologists (ACOG) and the Society for Maternal–Fetal Medicine support the US Preventive Services Task Force risk assessment and guideline criteria for the prevention of PE^1^, ^22^, ^23^. As per ACOG, all twin pregnancies are classified as being at high risk of developing GH/PE and should receive aspirin prophylaxis. Similarly, in the UK, the National Institute for Health and Care Excellence (NICE) classifies twin pregnancy as a moderate risk factor for PE and recommends aspirin prophylaxis if there is more than one moderate risk factor or one high risk factor^24^. Studies of singleton pregnancies have highlighted poor compliance with treatment and suboptimal performance of screening for PE when following NICE guidelines (detection rates of 30.4% and 40.8% for all PE and PE before 37 weeks' gestation, respectively, at a screen‐positive rate of 10.3%)^25^ and ACOG guidelines (detection rates of 5% and 2% for PE before 37 weeks' gestation and term PE, respectively, at a 0.2% false‐positive rate)^26^.

Modeling approaches that have been used in the prediction of HDP and GDM include logistic regression, competing risks and machine learning, using a combination of maternal characteristics and medical history, and biophysical and biochemical markers. Existing models based on logistic regression or a competing‐risks approach have not been validated for the prediction of HDP in twin pregnancy, because of poor calibration and overall poor fit^27^, ^28^, ^29^. It has also been suggested that these existing models may not apply to twin pregnancy because of modified biomarker profiles relative to singletons, as well as the need to use new distributions of biophysical and biochemical markers according to gestational age at delivery with PE^27^, ^28^.

In 2021, Benkő *et al*. investigated the prediction of PE in twin pregnancy at 11–13 weeks' gestation using a competing‐risks model incorporating maternal factors and serum biomarkers from two prospective multicenter datasets including 3938 pregnancies, of which 339 (8.6%) developed PE^27^. They found that the best performance of screening for PE was achieved by a combination of maternal factors, mean arterial pressure, uterine artery Doppler and placental growth factor. The detection rate for screening using maternal factors alone, at a 10% false‐positive rate, was 30.6% for delivery with PE < 32 weeks' gestation, with a corresponding AUC of 0.702 (95% CI, 0.622–0.782), which is close to that of our model. For the prediction of PE < 37 weeks' gestation, the detection rate, at a 10% false‐positive rate, was 24.9%, with an AUC of 0.742 (95% CI, 0.710–0.773).

Of the risk factors assessed in the present study, obesity had the highest importance on the Shapley additive explanation graph and, in the case of prepregnancy obesity Class II and III, was associated with more than double the risk of HDP and GDM. The pathophysiological changes underpinning obesity‐related cardiovascular risk, such as insulin resistance, hyperlipidemia, heightened state of systemic inflammation and oxidative stress, may be responsible for the increased incidence of HDP and GDM in pregnant women with obesity, as these factors can also affect placental development and function^30^, ^31^, ^32^, ^33^. In the prospective study of Solomon *et al*. of 14 613 singleton pregnancies, high maternal BMI (≥ 30 kg/m^2^) and maternal age were identified as significant risk factors for GDM^7^, which is consistent with our findings. However, unlike their study, which reported higher risks for GDM across various non‐White ethnicities, we found no effect and reduced odds of GDM in Hispanic and non‐Hispanic Black populations, respectively, potentially due to our larger sample size and more comprehensive adjustments for confounding variables.

Our study showed a protective effect of a lower education level (< 12^th^ grade) on HDP (aOR, 0.90 (95% CI, 0.87–0.92); *P* < 0.001). This contrasts with other studies showing that lower education level increases the risk of gestational hypertension^34^, ^35^, ^36^, ^37^. Our finding may reflect the influence of other factors related to lower education level, such as maternal birthplace outside the USA, which is associated with a lower risk of HDP; this so‐called ‘healthy immigrant’ effect typically results in health advantages for foreign‐born women^38^.

### Clinical and research implications

The findings of this study support using maternal risk factors for HDP and GDM to guide stratification and counseling in twin pregnancy, highlighting the potential for preconception changes in modifiable factors, particularly BMI. Lifestyle interventions, such as prepregnancy weight management, aimed at high‐risk groups can significantly lower the risk of HDP and GDM. The external performance of these prediction models is important, and future studies should seek to validate our findings. The advantage of machine learning algorithms is that the resulting calculators can be introduced easily and rapidly in an automated way, using cloud‐based or other online tools.

### Strengths and limitations

To the best of our knowledge, this is the largest national study to evaluate the association of HDP and GDM in twin pregnancy with simple and universally accessible maternal characteristics. Large sample sizes for the development of machine learning and logistic regression models and a low number of missing records are the main strengths of our study. Using both logistic regression and machine learning allowed us to construct a calculator to predict these conditions in twin pregnancy and to compare the performance of the two modeling approaches.

Our study has several limitations that should be acknowledged. We could not ascertain the impact of aspirin intake on the predictive performance of our models, which may have led to a spurious decrease in their predictive performance. A number of risk factors that are known to be associated strongly with the development of HDP, such as history of GH or PE in a previous pregnancy, chronic hypertension, chronic renal disease and systemic lupus erythematosus, were not available in the CDC dataset and, therefore, could not be incorporated into the models. Similarly, some risk factors that are known to be associated with the development of GDM, such as GDM in a previous pregnancy, type of treatment (diet or insulin), previous delivery of an infant with birth weight ≥ 4000 g, family history of diabetes and history of polycystic ovary syndrome, were lacking from the CDC dataset and could not be included in the analysis. Similarly, we could not analyze the association of chorionicity in our models, as relevant data were not available. Another limitation is the lack of testing of the prediction algorithm in other populations; we anticipate that adjustments would need to be made to the algorithm when applied to other populations.

### Conclusions

Our study demonstrates the utility of a novel automated machine learning approach using low‐cost predictors to estimate the risk of HDP and GDM in women with twin pregnancy. The predictive performance of the machine learning model is comparable to that of the logistic regression model. Clinical risk‐scoring approaches are used currently for risk assessment, but our findings suggest that machine learning could improve predictive accuracy and personalize care.

## Data Availability

Data available upon request to the corresponding author.
